# The analysis of Modified Qing’ E Formula on the differential expression of exosomal miRNAs in the femoral head bone tissue of mice with steroid-induced ischemic necrosis of femoral head

**DOI:** 10.3389/fendo.2022.954778

**Published:** 2022-08-10

**Authors:** Wei Zhu, Faxue Zhang, Junjie Lu, Chen Ma, Lin Shen, Desheng Hu, Xiaojuan Xu, Bo Shuai

**Affiliations:** ^1^ Department of Integrated Traditional Chinese and Western Medicine, Union Hospital, Tongji Medical College, Huazhong University of Science and Technology, Wuhan, China; ^2^ Department of Occupational and Environmental Health, School of Public Health, Wuhan University, Wuhan, China

**Keywords:** steroid-induced ischemic necrosis of femoral head, Modified Qing’ E Formula, transcriptome sequencing, microRNAs, bioinformatic analysis

## Abstract

**Objective:**

To investigate the differential expression of exosomal miRNAs in the bone marrow tissue of Modified Qing’ E Formula (MQEF) on steroid-induced ischemic necrosis of the femoral head (INFH) model.

**Methods:**

Steroid hormones were used to establish the INFH model and treated with MQEF. After successful modeling, femoral tissue exosomes were isolated for miRNA sequencing to obtain femoral tissue exosomal differential miRNAs. By GO analysis and KEGG analysis of the differential genes in both groups, the major exosomal miRNAs of MQEF exerting anti-INFH as well as the major signaling pathways were identified. Next, a quantitative metabolomic validation of MQEF with broad targeting was performed to obtain the main active components of MQEF and to perform biological analysis and signaling pathway prediction of the active components by network pharmacology. Finally, the sequencing results were validated by using RT-qPCR. The results of miRNA sequencing were verified by double examination of network pharmacology and RT-qPCR, and the exosomal miRNAs regulated by the anti-INFH effect of MQEF and the specific signaling pathway of the effect were clarified.

**Results:**

A total of 65,389 target genes were predicted in the exosomes of two groups of mice, and 18 significant differentially expressed miRNAs were obtained, of which 14 were up-regulated and 4 down-regulated. GO enrichment analysis showed that these predicted target genes were enriched in 12371 biological processes, 1727 cell components, and 4112 molecular functions. KEGG analysis showed that the predicted miRNA target genes were annotated to 342 signal pathways, in which the highly enriched pathways closely related to bone metabolism were PI3K-Akt signal pathway, MAPK signal pathway, and Wnt signal pathway. The most significantly up-regulated miRNAs were miR-185-3p and miR-1b-5p and the most significantly down-regulated miRNAs were miR-129b-5p and miR-223-5p, of which the targeted genes were closely related to the PI3K-Akt signal pathway. MQEF aqueous decoction extract targeted metabolomics quantitatively combined with network pharmacology predicted targets also closely related to PI3K-Akt signaling pathway. Real-time quantitative PCR validation showed that miR-185-3p was up-regulated 7.2-fold and miR-129b-5p was down-regulated 2.2-fold in the treatment group, and the difference was significant (P < 0.05).

**Conclusions:**

MQEF can regulate exosomal miRNA expression in steroid-induced INFH models, miR-185-3p or miR-129b-5p/PI3K-Akt signal axis may be part of the mechanism of MQEF against steroid-induced INFH.

## Introduction

Ischemic necrosis of the femoral head (INFH) is a devastating disease with tremendous harm, causing a huge physical, psychological, and financial burden to the patients. Beside trauma, the inappropriate use of corticosteroids is considered to be the most common cause of non-invasive INFH (1, 2). For some diseases like systemic lupus erythematosus, long-term or substantial use of corticosteroids is necessary. While corticosteroids relieve the symptoms of the disease, they cause harm to the patients especially those with INFH. Without positive intervention, INFH will mainly affect the hip joint. Only a fraction of patients who use corticosteroids develop hormonal osteonecrosis owing to the differences in genetic susceptibility in addition to the dose and frequency of use of the drugs among other factors. Since INFH is a devastating condition, the necessity of early and active treatment is emphasized. The main interventions currently include conservative treatment with bisphosphonates, anticoagulants, vasodilators, statins, and biophysical modalities, as well as surgical treatment with core decompression and total hip replacement. In China, traditional Chinese medicine (TCM) treatment is also used as an effective intervention (3).

MicroRNAs (miRNAs) are a class of small non-coding RNAs about 22 nucleotides (nt) in length. They play important regulatory roles by targeting messenger RNA (mRNAs) through degradation or inhibiting the protein translation. MiRNAs regulate about 60% of the mammalian genes and are involved in a series of biological processes such as cell proliferation, differentiation, apoptosis, neurogenesis, and tumor development (4). Therefore, miRNAs are an important class of gene regulators in the living organisms. MiRNAs also play an important role in bone metabolism and bone homeostasis. Specific changes in miRNA transcript levels or miRNA secretion levels are associated with the development and progression of skeletal diseases (5). They also affect bone formation, bone resorption, and angiogenesis in bone tissue microcirculation by regulating key transcription factors in bone metabolism, such as Runt-related transcription factor 2(Runx2) and bone morphogenetic protein (BMP) (6). In addition, miRNAs are used as biomarkers in clinical practice because they play a key regulatory role in bone metabolism and bone homeostasis.

Exosomes are small vesicles of 40–200 nm in size that are secreted by cells. They can perform the function of intercellular communication and transfer of substances. They also target and regulate cell differentiation and proliferation through delivering proteins and RNA. Many studies have shown that exosomes can play a protective role in various tissues and organs (7, 8). Similarly, exosomes can regulate bone metabolism and thus affect bone homeostasis. On one hand, exosomes can regulate the differentiation of mesenchymal stem cells into osteoblasts and promote the proliferation and activity of osteoblasts. On the other hand, exosomes can regulate the maturation and activity of osteoclasts. In addition, exosomes can promote the release of pro-angiogenic factors, enhance angiogenesis, promote the proliferation of vascular endothelial cells and blood vessel formation, thus promote bone regeneration and growth (9, 10).

Modified Qing’ E Formula (MQEF) has been used for more than 1300 years in China as a treatment for lumbodynia. It is now being incorporated in the guidelines for the treatment of postmenopausal osteoporosis and non-traumatic INFH. Current studies have shown that MQEF can improve the microstructure of bone trabeculae and bone biomechanics (11), improve the femoral bone mineral density, decrease the release of inflammatory factors, and promote the femoral vascular microcirculation in patients with non-traumatic ONFN by modulating the levels of adiponectin, BMP, osteoprotegerin, and other key transcription factors the affect the bone metabolism (12). MQEF also exerts an effective protective effect against osteoporosis by enhancing BMP2 levels, promoting the differentiation of bone marrow mesenchymal stem cells (BMSC) to osteoblasts, and increasing serum osteosclerin levels in postmenopausal patients with osteoporosis (13). Given that miRNA, exosomes, and MQEF are involved in the regulation of bone metabolism and bone homeostasis, we hypothesized that MQEF is involved in the regulation of bone metabolism and bone homeostasis through regulating exosomal miRNAs to play a therapeutic role in INFH. The effect of MQEF on the exosomal miRNAs of femoral head bone tissue in mice with steroid-induced INFH has not been previously reported. The aim of this study was to analyze the differential expression of miRNAs in the exosomes of local femoral head bone tissue of mice with INFH upon MQEF treatment by transcriptome sequencing technology and bioinformatics analysis.

## Methods

### Formula of TCM decoctions

According to the original formula in the Dictionary of Chinese Medical Formulas, the MQEF consists of *Eucommia ulmoides* (960 g), *Fructus psoralease* (480 g), *Semen juglandis* (300 g), and *Allium sativum* (240 g). All herbs were washed and placed in a multifunctional extractor. The herbs were immersed in double distilled water (five-folds of their volume) for 2 h followed by boiling for 2 h. After filtering of the boiled decoction, the herbal residues were repeatedly boiled twice for 1 h each time and the resulting boiled decoction was filtered, combined with the first filtrate, and condensed to a thick paste (100%), which was then added in 95% ethanol (three-folds of its volume) under stirring. After standing for 24 h, the solution was filtered to recover the ethanol fraction, which was then concentrated to a decoction of 5 g/mL. The mice received a daily intragastric MQEF dose of 1.7 g/kg body weight (b.w.) diluted with distilled water. The MQEF dose calculation was done in accordance with the guidelines correlating the dose equivalents between humans and laboratory animals, based on the ratio of the body surface area.

### Preparation, grouping, and intervention of experimental animal model

**Figure 1:**
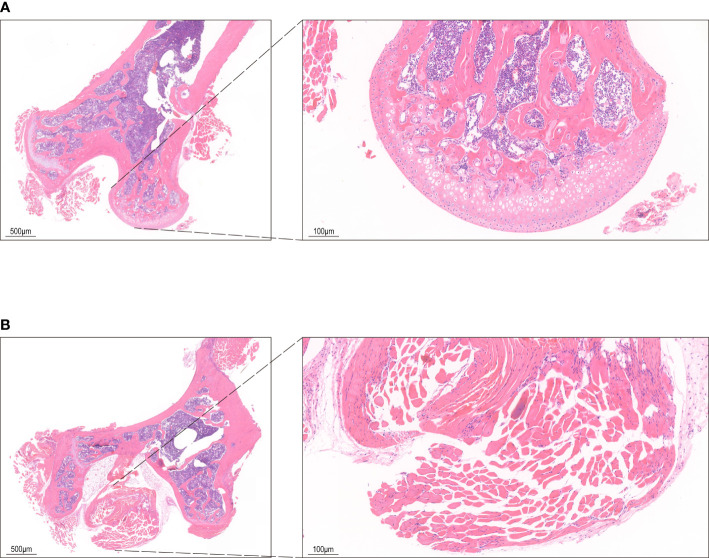
HE staining of bone tissue. **(A)** The control group shows bone cells in the bone lacunae, uniform nuclear staining and regular trabecular morphology. **(B)** HE staining in INFH group shows bone lacuna formation, sparse bone trabeculae, and disordered structure.

Sixteen male SPF C57BL/6 mice aged eight weeks (species name: Mus_musculus, source: NCBI, reference genome version: GCF_000001635.26_GRCm38.p6) were used to establish the steroid-induced INFH model according to the literature (14). The model was established by intravenous injection of lipopolysaccharide (LPS, Sigma, 10 µg/kg) for two consecutive days, followed by intramuscular injection of methylprednisolone (MPS, Pfizer, 20 mg/kg) for three consecutive days, with a 24-hour interval. One week later, four mice were randomly selected and sacrificed. The model was detected by HE staining (**Figure 1**). The remaining 12 mice were randomly divided into two groups, MQEF treatment group (n = 6) and placebo control group (n = 6), where the mice were treated with MQEF aqueous decoction extract and 200 µl normal saline once a day, respectively. The drug dosage was calculated according to the ratio of the body surface area between the humans and the mice. Three months later, the mice were sacrificed. The blood was taken from the heart and centrifuged. The bilateral femoral heads were carefully retrieved. Afterwards, the attached blood, muscles, and soft tissues were removed. Then, they were placed in a Petri dish containing 75% ethanol for 5 min, replaced with a new Petri dish containing 75% ethanol, and transferred to a clean bench. The ethanol-soaked femur was transferred to cold Dulbecco’s Phosphate-Buffered Saline (DPBS) (Servicebio, Wuhan, China) for soaking and washed until the ethanol on its surface was removed. After cutting off one end of the femur with scissors, a 1 mL syringe was used to aspirate cold DPBS to blow the bone marrow out of the femur, and the femur was repeatedly rinsed until no visible red color could be seen. Clumps of bone marrow cells in the DPBS were gently dissociated using a 5 ml pipette to form a single-cell suspension which were then sieved using a 70 µm cell filter (Biosharp, Hefei, China), transferred into a 15 mL centrifuge tube, centrifuged at 1500 rpm for 5 min, and the supernatant was discarded. Then, the cell pellets were resuspended in erythrocyte lysate for 5 min and centrifuged at 1500 rpm for 5 min. The supernatant was discarded and the pellets were stored in a -80°C freezer.

### Extraction of exosomes from bone tissue by ultracentrifugation

Exosomes from the bone marrow fluid were extracted by gradient ultracentrifugation (15, 16). The bone marrow fluid was evenly divided into 10 ml centrifuge tubes and PBS was added until the volume reached 10 ml. After centrifugation (Eppendorf centrifuge 5810R, Germany) at a speed of 300 g for 10 min at 4 °C, the supernatant was transferred to new 10 ml centrifuge tube and centrifuged at a speed of 2,000 *g* for 10 min at 4 °C. The supernatant was transferred into a centrifuge tube (10 ml) for ultracentrifugation and centrifuged at a speed of 12,000 *g* for 30 min at 4°C. Then, the supernatant was transferred into another centrifuge tube (10 ml) and ultracentrifuged (Optima XE, Backman Counter Life Science, USA) at a speed of 120,000 *g* for 70 min at 4°C. The transparent precipitate was retrieved and resuspended with 50 µl of PBS. The resulting exosome solution was stored at -80°C.

### The quality control of the extracted exosomes

The quality control of the extracted exosomes is essential. The International Society for Extracellular Vesicles suggests three levels of exosome identification: Scanning Electron Microscopy or Transmission Electron Microscopy to identify morphological features, Nanoparticle Tracking Analysis (NTA) to identify particle size and concentration, and protein markers.therefore, we have performed NTA (**Supplementary Figure 1A**) and western-blot assays (**Supplementary Figure 1B**) on exosome samples. We used the currently recognized and most used exosome-specific markers: CD63 and TSG101 for western blot assays identification. NTA is a single particle tracking technology combined with classical microelectrophoresis techniques (zeta potential) and Brownian motion, using the Stockes-Einstein equation to calculate the hydrodynamic diameter and concentration of nanoparticles. The results of these two identifications showed that the bone marrow exosomes isolated by ultracentrifugation were qualified and fulfilled the requirements of subsequent experiments.

Isolated exosomes were diluted in PBS and analyzed using the Nanoparticle tracking analyzer ZetaView (Particle Metrix, Germany) equipped with a green laser (520 nm). Nanoparticles illuminated by the laser and their movement under Brownian motion was captured. Videos were analyzed using the NTA software to provide particle concentrations and size distribution profiles. Triplicate measurements were recorded for each sample. Size distribution and concentration profiles were averaged across replicates to derive the representative size distribution profiles.

Isolated exosomes were lysed by RIPA Lysis Buffer (Servicebio, Wuhan, China) with the addition of Phenylmethanesulfonyl fluoride (Servicebio, Wuhan, China). Protein loading buffer was added and protein denaturation was carried out at 95 °C for 15 minutes. The protein samples were separated by 10% or 12% twelve alkyl sulfate polyacrylamide gel electrophoresis (SDS-PAGE) and transferred onto a PVDF membrane (Merckmilipore, Darmstadt, Germany). The PVDF membrane was blocked with 5% milk in TBST powder for 1 h and incubated with the primary antibody (CD63/TSG101, Servicebio, Wuhan, China) at 4°C overnight. The membranes were washed with TBST three times, then incubated with HRP conjugated secondary antibody at room temperature for 1.5 h. After being washed with TBST for three times, membranes were incubated with ECL solution (Biosharp, Hefei, China), and the images were obtained with iBright CL750 imaging system (ThermoFisher Scientific, Massachusetts, USA).

### Analysis regimen, sample correlation, and expression distribution

A total of 12 samples were measured in this study using the DNBSEQ platform and each sample generated an average of 39.76 M of data with an average matching rate of 91.4% of the reference genome among the samples. A total of 65,389 genes were detected, including 64,406 known genes and 983 unknown genes (**Supplementary Figure 2A**). Moreover, 734 miRNAs were detected. The box plot shows the distribution of gene expression levels of each sample, and the spread of data distribution can be observed (**Supplementary Figure 2B**). The stacked bar graph plot directly displays the number of genes in different TPM value ranges of each sample (**Supplementary Figure 2C**). The density plot depicts the trend of gene abundance as the expression level changed in the samples which clearly reflected the concentration range of gene expression in the sample (**Supplementary Figure 2D**). In addition, we performed association analysis between the detected genes and transcription factors (**Supplementary Figure 2E**).

### Filtering and quality control of sequencing data

The sequencing platform of DNBSEQ was used and the sequencing read length was 50 bp. The raw data of sequencing contained reads with low quality, linker contamination, and high unknown base (N) content. These reads needed to be removed before data analysis to ensure the reliability of the results. The quality indexes of filtered reads are shown in the quality statistics of reads after filtering (**Supplementary Figure 3A**). The statistics of filtered components of raw data (**Supplementary Figure 3B**), the distribution of base content of clean reads (**Supplementary Figure 3C**), and the base quality distribution of clean reads (**Supplementary Figure 3D**) are also shown.

### Number, classification, and length of small RNAs in each sample

The unique species and total number of sequences of small RNA (sRNA) were counted, and the length distribution of sequences of sRNAs was calculated (**Supplementary Figures 3E, F**). The length interval of sRNA is 18–30 nt, and the peak of length distribution can help us to judge the unique species of sRNAs. For example, miRNAs are concentrated in 21 or 22 nt, siRNAs are concentrated in 24 nt, while piRNAs are distributed in 28–30 nt. In addition, in most cases, there is an obvious difference in the sequence length distribution between plant and animal samples. Specifically, the peak of length distribution of plant samples generally appears in 21 nt or 24 nt, while that of animal samples appears in 22 nt. According to these conditions, some preliminary assessments can be made for the sample situation and sequencing condition.

### Widely targeted metabolomics quantitative verification of plants

About 150 mg MQEF aqueous decoction extracts were weighted and added to a 2 mL grinding thickened tube with 1 mL extract buffer (methanol: water=7:3, v:v, pre-cooled at -20°C) and two small steel balls. Then, samples were crushed using a tissuelyser for 5 min at 50 Hz. Samples were then stored at 4°C, vortexed 3 times every 10 minutes, and then incubated overnight at 4°C. Vortexing and centrifugation at 13,000 *g* was done for 10 minutes at 4°C. After centrifugation, 800 μL of the supernatant was filtrated with a 0.22-μm filter membrane before LC–MS analysis. The sample extracts were analyzed using UPLC (Waters, USA) equipped with QTRAP 6500 Plus (SCIEX, USA). Samples injected in the ACQUITY UPLC HSS T3 column (100*2.1 mm, 1.8 μm, Waters) were eluted for MRM monitoring with the following LC gradient: 95% mobile phase A (Waters with 0.1%FA) and 5% mobile phase B (Acetone with 0.1%FA) at 0 min to 5% mobile phase A and 95% mobile phase B in 22 min with the flow rate at 0.3 mL/min and the column temperature was set at 40°C. For the QTRAP 6500 Plus equipped with an EST Turbo Ion-Spray interface, the source parameters were set as follows: source temperature: 500°C; ion spray voltage (IS): 4500 V (positive mode) or -4500 V (negative mode); Ion source gas I (GS1), gas II (GS2), and curtain gas (CUR) were set at 40, 40, and 20 psi, respectively. MRM methods were set at schedule mode with MRM transitions, collision energy (CE), declustering potential energy (DP), and retention time for target metabolites (**Supplementary Figure 4**).

### Screening of active ingredients and enrichment by KEGG

The top 50 ingredients (accounting for 79.8% of the total content) were analyzed in order of content. Firstly, the Canonical SMILES numbers of 50 compound ingredients were obtained through the PubChem database, and the Canonical SMILES numbers were entered into the Swiss ADME web tool. The potential active chemical ingredients (top 50 in content) of the compounds were evaluated according to Lipinski’s “Rule of Five.” Thirty-four active chemical ingredients were obtained and the molecules which were not suitable to be drugs were excluded by screening. The Canonical SMILES numbers of the 34 active chemical components were entered into the Swiss Target Prediction database and their potential action targets against Homo sapiens were predicted according to their chemical structures. A total of 597 predicted targeted genes were obtained after de-duplication. Next, KEGG enrichment analysis was carried out on the 597 predicted targeted genes.

### Microarray analysis

The expression profiling by array of steroid-induced INFH (GSE123568) was downloaded from Gene Expression Omnibus (GEO) database. We used the R package of GEO query (version 2.54.1) for the data download, limma (version 3.42.2) for differential analysis, umap (version 0.2.7.0) for UMAP analysis, ggplot2 (version 3.3.3) for visualization, and ComplexHeatmap (version 2.2.0) for heatmap visualization. Based on data from 30 patients with INFH and 10 patients with non-INFH, gene expression profiles were detected by microarray analysis. Then, the list of candidate gene biomarkers for INFH was identified by integrating differential expression data analysis and gene signaling transduction network analysis.

### Construction of PPI network and calculation of rank value

PPI networks were constructed using the String database (v 11.5) for each of the 62 targets involved in the PI3K-Akt signaling pathway and 354 pathway targets. The degree values were calculated using Network Analysis (a Cytoscape plugin). The nodes with the top 20 degree values in the 62 targets involved in the PI3K-Akt signaling pathway were called core targets, which were then matched with the rank in the network of 354 pathways. Then, the targets of 34 active chemical ingredients of MQEF were intersected with the 62 targets involved in PI3K-Akt signaling pathway to explore which ingredients were involved in regulating PI3K-Akt signaling pathway in MQEF. Finally, a total of 23 active ingredients targeted at the 62 targets were involved in the pathway.

### Intestinal absorption rate, drug-likeness properties, and safety assessment

First, the CAS number and Canonical SMILES number of the top 50 ingredients in MQEF were obtained from the PubChem database and the molecular weight (MV) was also acquired. Then, the TCMSP database was used to retrieve the top 50 MQEF ingredients and their intestinal absorption rate, Caco2, and Drug Likeness. Some ingredients were not included in the TCMSP database. Therefore, the input intestinal absorption rate and bioavailability score were evaluated by Swiss ADME database. Caco2 permeability or intestinal absorption rate were used to assess the possibility of ingredients becoming drugs. Finally, we used Pro-ToxII database to estimate the median lethal dose (LD50) of 50 ingredients.

### Verification of sequencing results by RT-qPCR

The exosomal miRNA was extracted with RNA Isolate (Vazyme, MiPure Cell miRNA Kit, Nanjing, China) and Trichloromethane (Sinopharm Chemical Reagent Co., Ltd., Shanghai). MiRNAs were obtained through the MiPure miRNA Column according to the manufacturer’s protocol (Vazyme, MiPure Cell miRNA Kit, Nanjing, China). The miRNA 1st Strand cDNA Synthesis Kit (by stem-loop) (Vazyme, MiPure Cell miRNA Kit, Nanjing, China) was used for genomic DNA removal and cDNA acquisition. Then, the cDNA was amplified using the fluorescence quantitative PCR instrument (ABI StepOnePlus Real-Time PCR System with Tower, Massachusetts, USA). Amplification reactions were performed according to the manufacturer’s protocol (Vazyme, miRNA Universal SYBR qPCR Master Mix, Nanjing, China) under the reaction conditions used for amplification. The calculation is based on 2^−ΔΔCt^ method and β- actin was used as a reference to normalize the expression and calculate the relative expression of each group. The miRNA sequences are as follows: mmu-miR-185-3p: (5’→3’): F: GAGGCTGGAGCTCTCAGGCCACCTGCCCAGGGCGACTCCC; R: GGGAGTCGCCCTGGGCAGGTGGCCTGAGAGCTCCAGCCTC; mmu-miR-129b-5p: (5’→3’): F: CTTTTTGCGGTCTGGGCTTGC; R: AGTGCAGGGTCCGAGGTATT.

### Statistical analysis

All data were presented as means ± SEM of three independent experiments and analyzed using one-way ANOVA combined with Tukey’s multiple comparison tests. P < 0.05 was considered statistically significant.

## Results

### Analysis of differential expression of miRNAs in bone tissue exosome of the two groups

**Figure 2:**
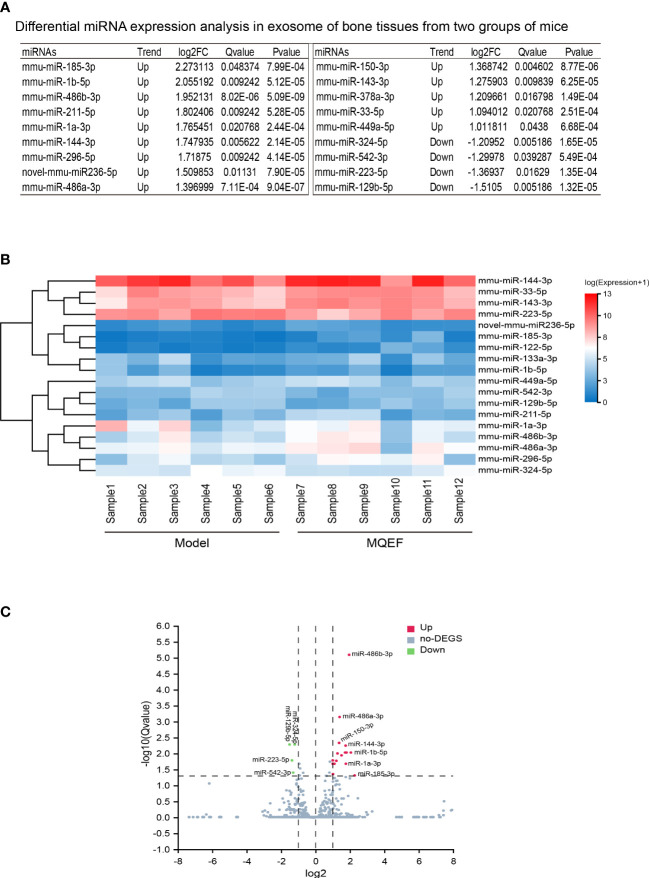
Differential gene display. **(A)** Differential miRNAs in bone tissue exosome sorted by LogFc. We ranked the measured miRNAs in the priority order of MiRbase > pirnabank > snoRNA (human/plant) > Rfam > other sRNA for annotation, and if none of the above databases were retrieved, they were defined as novel miRNA. We defined the significant difference threshold as log2FC>=1 and Q-value<=0.05. Based on statistical analysis, a total of 18 miRNAs expression was defined having a difference between the two groups; **(B)** Differential gene heat map, the horizontal axis is log2 (expression value +1) of the sample, and the vertical axis is the gene. The redder the color of the color block the higher the expression, the bluer the color the lower the expression. **(C)** Differential gene volcano plot, the X-axis represents the log2-transformed differential fold value, and the Y-axis represents the -log10-transformed significance value. Red represents up-regulated DEGs, blue represents down-regulated DEGs, and gray represents non-DEGs.

There were 18 significant differentially expressed miRNAs in the exosomes of femoral head bone marrow tissue in the treatment group and the control group, of which 14 were up-regulated and 4 were down-regulated. We defined the significant difference threshold as log2FC>=1 and Q-value<=0.05. Based on the statistical analysis, the statistically significant results obtained are presented in the figure as follows (**Figures 2A–C**).

### GO enrichment analysis of predicted target genes of differentially expressed miRNA

**Figure 3:**
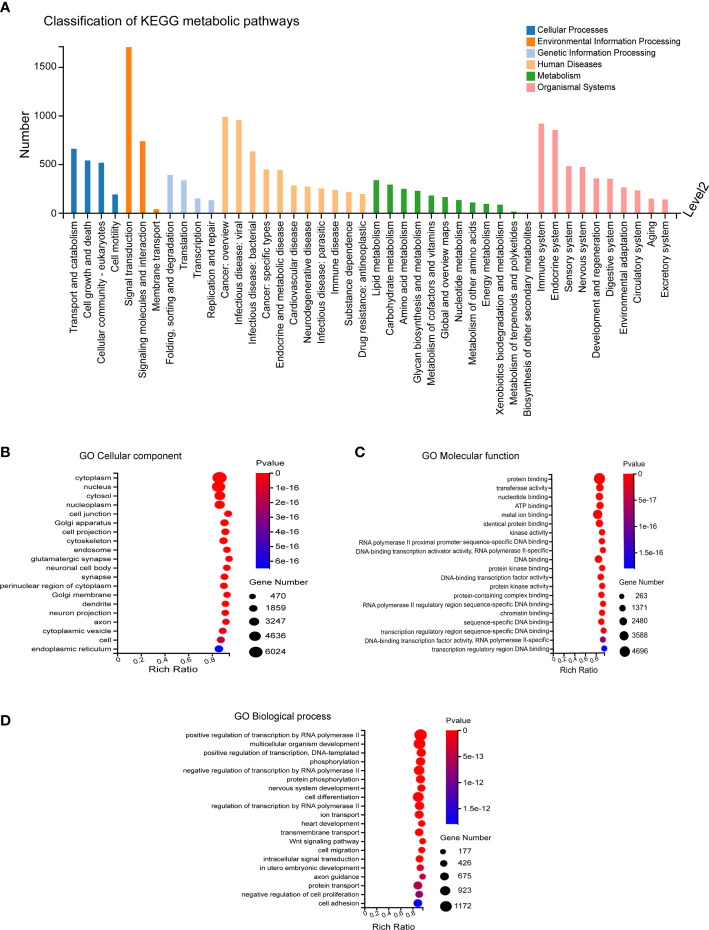
GO Enrichment. **(A)** Biological processes involved in the differentially expressed miRNA-regulated predicted target genes. **(B–D)** Cellular components, molecular functions and biological processes involved in the differentially expressed miRNA-regulated predicted target genes.

The predicted target genes regulated by differentially expressed miRNAs mainly participated in biological processes, including cellular process, biological regulation, regulation of biological process, metabolic process, and response to stimulus. The involved cellular components mainly included cell, cell part, organelle and membrane, and the associated molecular functions mainly included binding, catalytic activity, and molecular function. These predicted target genes were enriched in 12,371 biological processes, 1,727 cell components, and 4,112 molecular functions (**Figures 3A–D**).

### KEGG enrichment analysis of predicted target genes of differentially expressed miRNA

**Figure 4:**
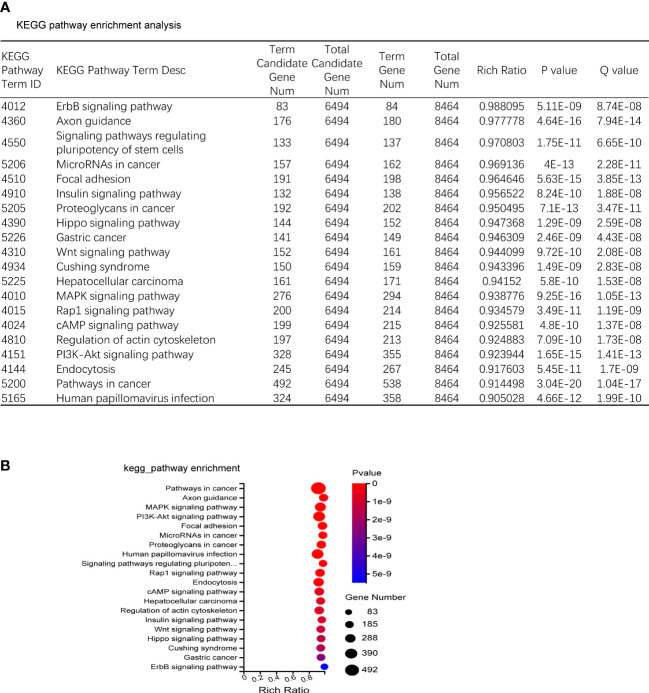
KEGG enrichment. **(A)** KEGG pathway sorted by enrichment ratio; Rich Ratio = Term Candidate Gene Num/Term Gene Num. **(B)** Enrichment bubble diagram.

The predicted target genes of miRNAs were annotated to 342 signal pathways, among which the highly enriched pathways closely related to bone metabolism were PI3K-Akt signal pathway, MAPK signal pathway, and Wnt signal pathway. The most significantly up-regulated miRNAs were miR-185-3p and miR-1b-5p. While the most significantly down-regulated miRNAs were miR-129b-5p and miR-223-5p, of which the targeted genes were closely related to the PI3K-Akt signal pathway and Wnt signal pathway (**Figures 4A, B**).

### Widely targeted metabolomics quantitative verification of MQEF and verification of sequencing results by network pharmacology

**Figure 5:**
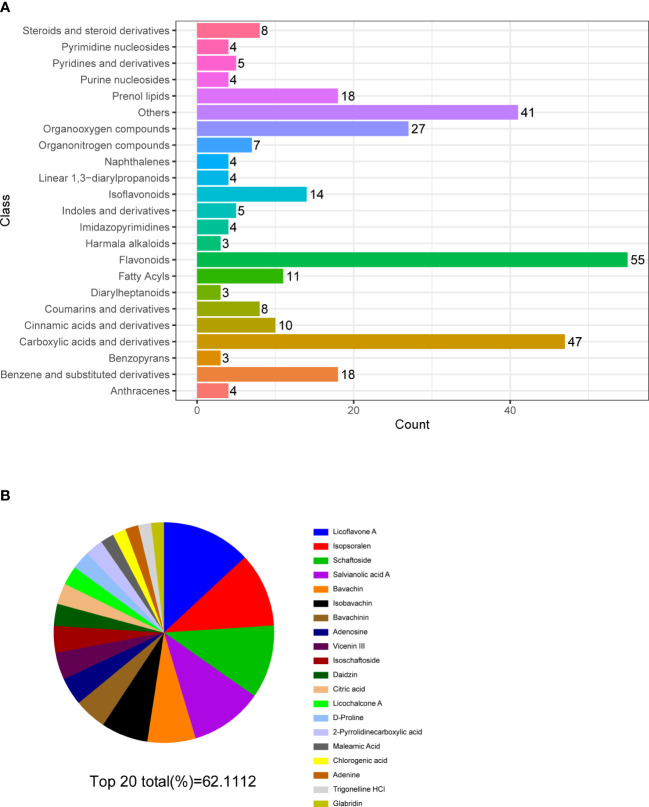
Multiple reaction monitoring. **(A)** Classification and functional annotation of metabolites identified by LC/MS of MQEF aqueous decoction extract. **(B)** The top 20 small molecules in terms of relative percentage of each metabolite component calculated by the peak area normalization method.

**Figure 6:**
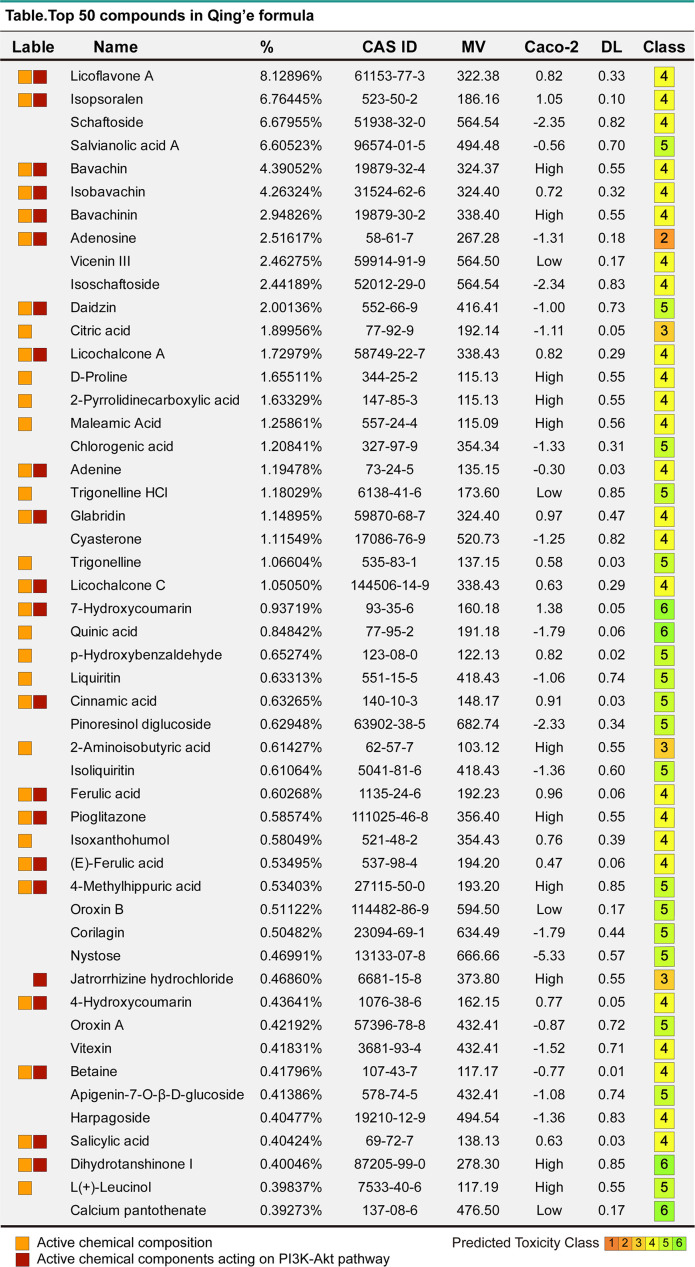
The top 50 compounds of MQEF. The top 50 compound names, percentages, CAS numbers, molecular masses MV, oral absorption and utilization Caco2, drug-like DL and toxicity class assessment (1-6, toxicity minimum 6) of MQEF.

**Figure 7:**
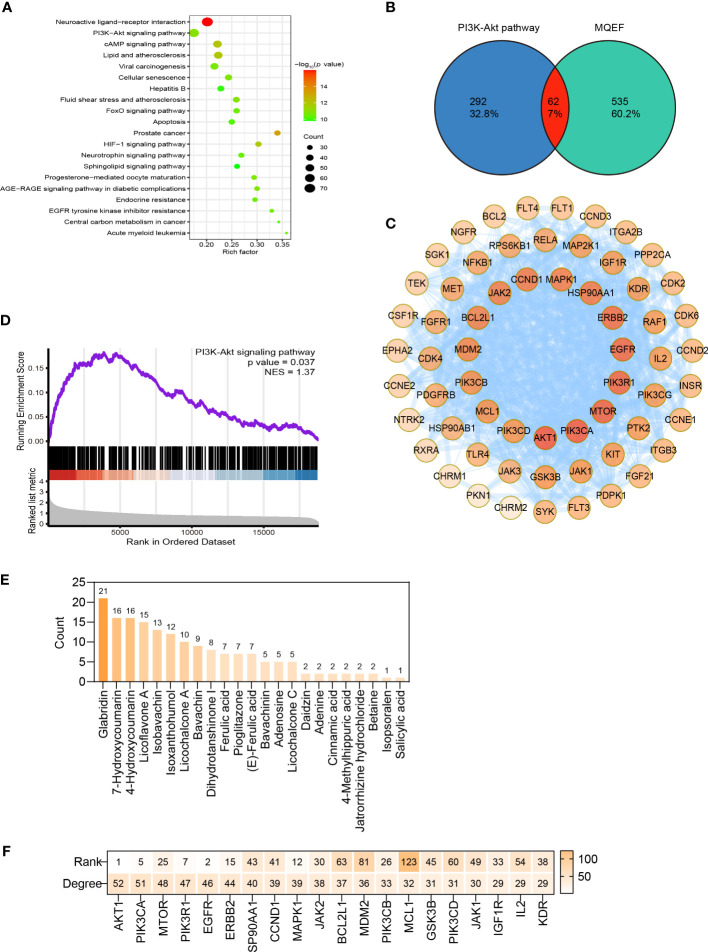
Network pharmacology. **(A)** Top 20 KEGG enrichment pathway for the 597 targets of the 34 active ingredients in MQEF. **(B)** The intersection of 354 targets in the PI3K-Akt pathway with 597 targets in the MQEF were obtained by drawing Venn diagrams from the R package (Venn Diagram). **(C)** The 62 key targets for MQEF to exert anti-INFH through the PI3K-Akt signaling pathway. **(D)** Gene expression profile of the PI3K-Akt signaling pathway found to be significantly enriched in hormonal femoral necrosis disease by microarray analysis. **(E)** Degree of the role of the 62 targets in the PI3K-Akt signaling pathway. **(F)** The 34 active chemical components of the MQEF were intersected with 62 intersecting targets involved in PI3K-Akt signaling pathway to explore which compounds have more targets to regulate PI3K-Akt signaling pathway, and finally there were 23 active components corresponding to 62 targets involved in the pathway.

**Figure 8:**
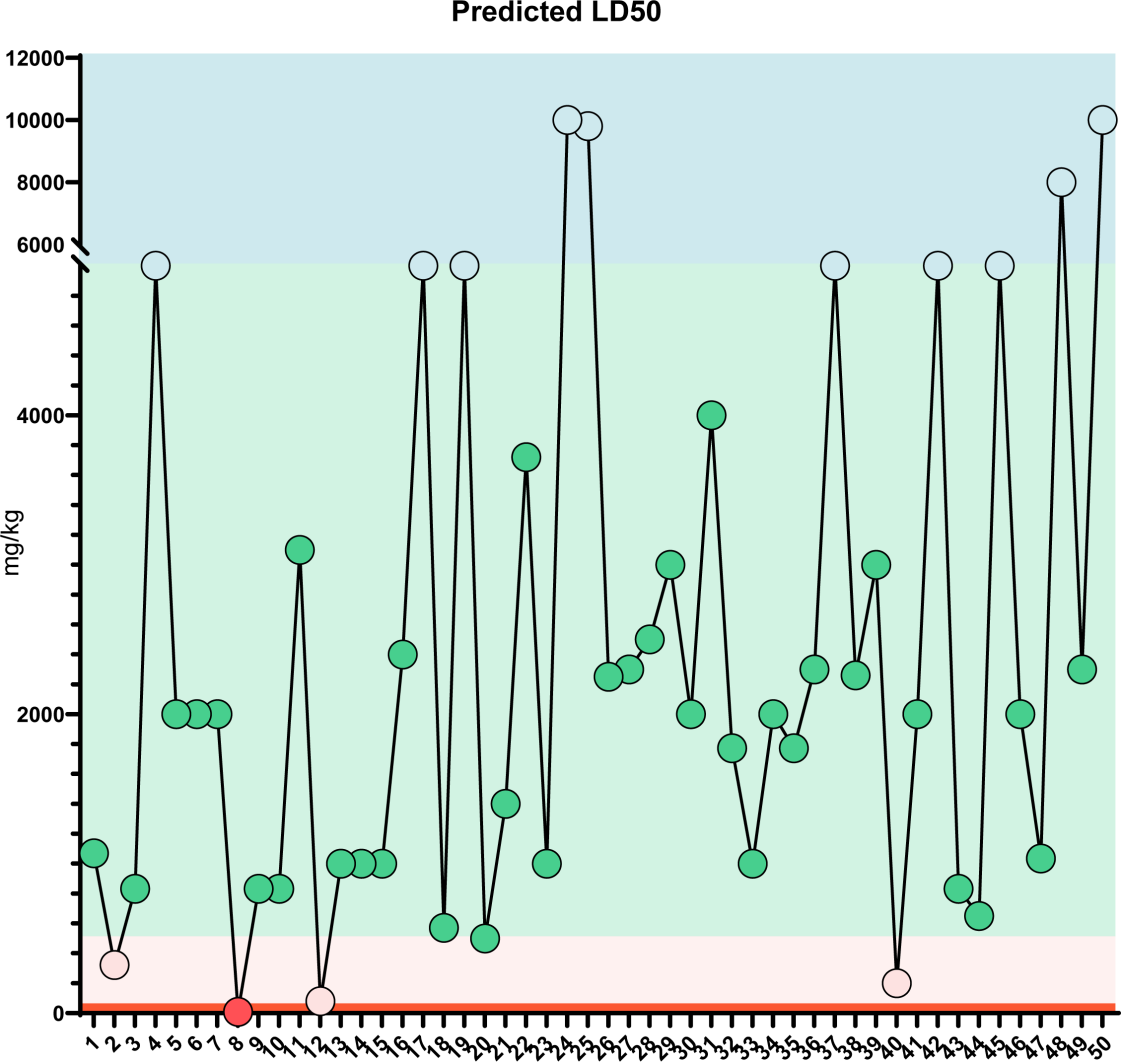
LD50 prediction: Predicted LD50 toxicity for the top 50 compounds of MQEF. The horizontal coordinates, from 1 to 50, are the top 50 compounds ranked according to the percentage of Modified Qing’ E Formula aqueous decoction extracts obtained from plant metabolomics, and the vertical coordinates indicate the values of LD50 obtained by database prediction in 4 classes of mg/kg, which are distinguished by four different colors.

In order to verify the accuracy of the sequencing results, we used widely targeted metabolomics quantitative verification and network pharmacology to verify the sequencing results. Firstly, LC–MS/MS technique was used to identify and quantitatively analyze the MQEF decoction extract. A total of 571 ingredients were identified, of which flavonoids accounted for the highest proportion followed by carboxylic acids. We displayed the top 20 ingredients (**Figures 5A, B**) and then predicted the oral availability, drug-likeness properties, and toxicity of the top 50 ingredients. The 34 active ingredients were screened out (**Figure 6**) according to Lipinski’s “Rule of Five.” The 34 active chemical components with Canonical SMILES numbers were entered into the SwissTargetPrediction database (http://www.swisstargetprediction.ch/) to predict their potential targets of action against Homo sapiens based on their chemical structures. After summarization and de-duplication in an Excel spreadsheet, a total of 597 active ingredient targets were obtained (**Supplementary Table 1**). To find the potential process that the 597 predicted targets are involved in, we next performed enrichment analysis by KEGG and found that the predicted acitve targets of MQEF were concentrated in PI3K-Akt signaling pathway (hsa04151) (**Figure 7A**). Then, the intersection of 354 targets (**Supplementary Table 2**) in the PI3K-Akt pathway with 597 targets in the MQEF were obtained by drawing Venn diagrams from the R package (Venn Diagram) (**Figures 7B, C**). These 62 targets could be key targets for MQEF to exert anti-INFH through the PI3K-Akt signaling pathway (**Supplementary Table 3**). To explore whether PI3K-Akt signaling pathway plays an important role in steroid-induced INFH, we used microarray analysis to detect gene expression profile (**Figure 7D**). It was found that PI3K-Akt signal pathway was significantly enriched in steroid-induced INFH. By inducing the activation of PI3K-Akt signal pathway, steroid-induced INFH can be significantly improved (17–19). In order to explore whether the 62 targets of MQEF were the key targets of PI3K-Akt signal pathway. The 62 targets obtained above were used to construct the PPI interaction network and calculate the degree values. It was found that the 34 active ingredients of MQEF acted on the core targets of the 62 targets in PI3K-Akt signal pathway (**Figures 7E, F**). In conclusion, the PI3K-Akt signaling pathway plays an important role in steroid-induced INFH, MQEF has the most significant and quantitative effect on the PI3K-Akt signaling pathway, and MQEF acts on key targets of the PI3K-Akt signaling pathway. We also predicted the toxicity and found that most of the ingredients in MQEF were assessed as mild toxicity, and their LD50 ranged from 500 to 5,000 mg/kg (**Figure 8**), with no carcinogenic, teratogenicity, or mutagenicity. Some small molecular ingredients may have mild hepatotoxicity, so the MQEF need to be reasonably used.

### Verification of sequencing results by RT-qPCR

**Figure 9:**
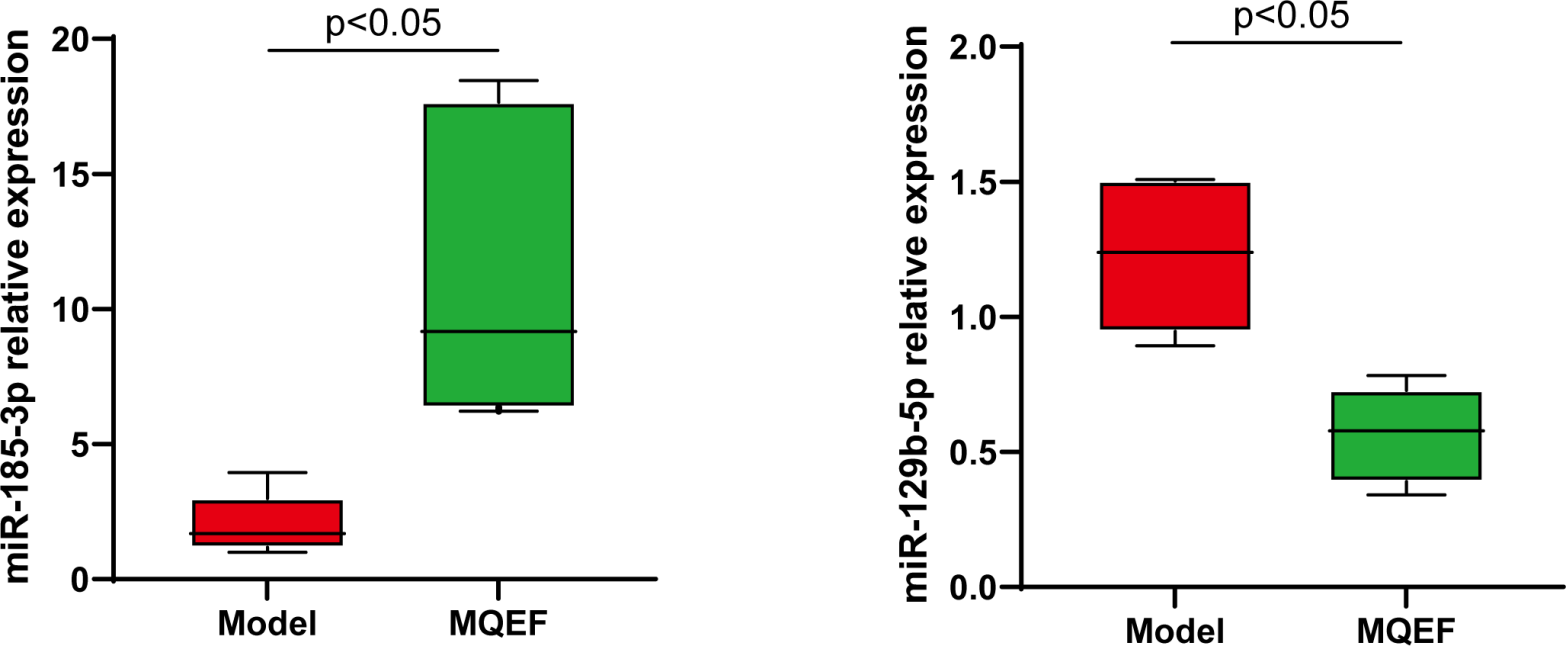
RT-QPCR analysis of bone marrow exosome miRNA, and the difference was significant (P < 0.05).

To further verify the accuracy of the sequencing results, the most significant up-regulated and down-regulated miRNAs from the above ultimate sequencing analysis were selected for RT-qPCR. Both miR-185-3p and miR-129-5p were included in the experiment. The total RNA samples of the exosomes of the femoral head from the two groups were measured repeatedly for 3 times. The relative expressions of miR-185-3p and miR-129-5p were detected by RT-qPCR. Results showed that miR-185-3p was up-regulated by 7.2 times in the experimental group and miR-129-5p was down-regulated 2.2 times in the experimental group with significant differences (P < 0.05) (**Figure 9**). The results of RT-qPCR and miRNA sequencing were consistent indicating that the above results of high-throughput sequencing were credible.

## Discussion

INFH caused by the inappropriate use of corticosteroids currently presents many therapeutic challenges. Therefore, exploring the mechanisms that can regulate bone metabolism and bone homeostasis can provide the theoretical basis and frontier discoveries for steroid-induced INFH (20). The two main areas of research to promote bone regeneration are biomaterials and cellular therapy, both of which have some disadvantages to different degrees. The toxicity of biomaterials and the resulting immune rejection can lead to severe harm to the patients, while the uncontrollable characteristics of cellular therapy can lead to tumor transformation and unacceptable outcomes (21, 22). In recent years, it has been discovered that exosomes are small vesicles that perform intercellular functions of intercellular communication and transfer of substances. By delivering lipids, proteins, or RNA, exosomes can regulate the biological activity of cells. Exosomes have a better safety profile and a more powerful ability to promote bone reproduction. This has shed new dawn on the treatment of steroid-induced INFH. The current study revealed that exosomes directly regulates MSC differentiation toward osteogenesis. Moreover, exosomes secreted by differentiated osteoblasts further activate the Wnt signaling pathway and promote osteogenic differentiation of MSC, thus establishing a positive feedback regulation of osteogenic signaling (23).In addition, exosomes can directly regulate osteoblast activity and proliferation to promote bone regeneration. Along with the regulation of direct skeletal-related cells, exosomes also have a stimulatory effect on endothelial cells, which can promote endothelial cell proliferation, migration, and angiogenesis, which may be beneficial to improve the local vascular microcirculation disorders and insufficient blood supply in INFH. The activation of angiogenesis is one of the favorable conditions to stimulate bone growth (24). Compared with biomaterials and cell therapy, exosome therapy has its unique advantages. Exosomes are secreted and released by autologous cells, and therefore do not cause immune rejection and toxicity unlike biomaterials. Compared with live cell therapy, the vesicles themselves are not active and their risk of causing an increased chance of tumor formation will be greatly reduced. Furthermore, exosomes can be preserved for a longer period of time *in vitro* and have good stability.

Exosomes deliver various molecules, among which miRNAs are the first to enter exosomes and are preferentially transported and delivered by exosomes. MiRNAs regulate various physiological and pathological processes by targeting mRNAs for degradation or inhibiting protein translation. By targeting mRNAs and proteins that regulate bone metabolism, miRNAs exert regulatory effects on the complex signaling pathways involved in bone metabolism through these molecules, thereby further regulating cartilage and osteogenic differentiation. Many “star” molecules and pathways involved in bone metabolism, bone homeostasis, and bone reconstruction, such as BMP2, RUNX2, and Wnt signaling pathways, are regulated by miRNAs (25). For example, RUNX2, a transcription factor, is essential for osteoblast differentiation and cartilage formation. RUNX2 is abundantly expressed in osteoblast precursor cells, osteoblasts, chondrocytes, and other bone-related cells (26). RUNX2 activates multiple signaling pathways and regulates the proliferation and differentiation of chondrocytes and osteoblasts. Several miRNAs, such as miR-23a, miR-30 family, and miR-133, regulate chondrocyte or osteoblast activity differentiation and proliferation by targeting RUNX2, either by up-regulating or down-regulating its expression (27). Thus, miRNAs play an important role in bone metabolism, bone homeostasis, and bone reconstruction. Thus, regulating this small non-coding RNA can be a favorable therapeutic approach for INFH.

In China, MQEF is used for the treatment of postmenopausal osteoporosis and non-traumatic femoral head necrosis and has received positive responses (28). The use of MQEF is based on Chinese medical theory and lacks modern pharmacological studies. The molecular mechanism has not yet been clarified. As a result, there are restrictions on the use of MQEF. In recent years, this situation is changing. An increasing number of studies on the molecular mechanism of MQEF are being published. MQEF aqueous decoction contains natural phytoestrogens with high affinity for estrogen receptor α (ERα), which can produce estrogen-like therapeutic effects, and due to the form of the formula, it creates a synergistic effect that produces maximum estrogenic activity while mitigating adverse effects (29). MQEF improves bone trabecular structure and bone biomechanics in estrogen-deficient mice by increasing β-catenin expression in the Wnt pathway (11), promotes differentiation of BMSCs into osteoblasts, and increases serum osteosclerin levels in postmenopausal patients with osteoporosis. The patients with non-traumatic femoral head necrosis can effectively increase bone mineral density, reduce inflammation levels, improve local hypercoagulability and hemodynamic levels by increasing serum adiponectin and BMP2 expression levels by using MQEF (12). The clinical efficacy of MQEF is obvious, but whether MQEF can regulate the level of exosomal miRNA has not been reported yet, and whether the mechanism of MQEF ‘s therapeutic effect is to regulate the expression of target genes by targeting exosomal miRNA, and to play a regulatory role in the upstream and downstream signaling pathways involved in bone metabolism is not known yet. In addition, the specific composition of the small molecule compounds contained in MQEF as a formula has not been elucidated yet, which are worth exploring.

In this study, we first conducted exosome isolation and miRNA sequencing in an animal model of steroid-induced INFH with MQEF intervention. Through transcriptomic studies, we clarified that one of the mechanisms by which MQEF exerts its therapeutic effects on steroid-induced INFH is by targeting exosomal miRNAs and through up-regulation and down-regulation of some miRNAs, which regulate multiple signaling pathways involved in the upstream and downstream of bone metabolism, such as The predicted target genes of MQEF-regulated exosomal miRNAs were annotated to a total of 342 signaling pathways, and the highly enriched pathways were PI3K-Akt signaling pathway, MAPK signaling pathway and Wnt signaling pathway in order. There were 328 predicted target genes annotated to PI3K-Akt signaling pathway, 276 predicted target genes annotated to MAPK signaling pathway, and 152 predicted target genes annotated to Wnt signaling pathway. Some of the predicted target genes regulated by the differentially expressed miR-185-3p, miR-486-3p, and miR-542-3p were annotated to PI3K-Akt signaling pathway, MAPK signaling pathway, and Wnt signaling pathway, therefore it is speculated that miR-185-3p, miR-486-3p and miR-542-3p in the local exosome of bone tissue miR-542-3p may play an important role in the pathological process of MQEF intervention in INFH. We also analyzed the active ingredients of MQEF aqueous decoction extract for the first time by metabolomics and clarified the distribution and proportion of small and medium molecule compounds in MQEF. Further, we verified the sequencing results by network pharmacology, and found that the PI3K-Akt signaling pathway was highly correlated with INFH, and the MQEF active ingredients significantly affected the PI3K-Akt signaling pathway and acted on the key targets of the PI3K-Akt signaling pathway.

Next, we selected two key differentially expressed miRNAs and investigated how miRNAs regulate hormonal INFH through key signaling pathways of bone metabolism, especially PI3K-Akt signaling pathway, Wnt signaling pathway, and MAPK signaling pathway. We also searched for specific effective components in MQEF that play a role in the treatment of steroid-induced INFH at the molecular level to provide strong theoretical evidence for exploring the modern pharmacological mechanism and standardizing the use of MQEF. In addition, MQEF is composed of many small molecules, and although its clinical efficacy in the treatment of steroid-induced INFH is confirmed, yet it is unknown which specific small molecules are responsible for the effect and the specific mechanism, which deserves further investigation. Furthermore, many small molecule compounds in MQEF have disadvantages such as poor oral bioavailability, thus limiting their availability. However, to date, few studies have been conducted and no feasible solutions have been proposed. In order to use MQEF in a more standardized and scientific way in the future, great efforts are needed to optimize the ratio, dosage form, and delivery method of MQEF.

## Conclusions

In conclusion, this study clarified that one of the mechanisms by which MQEF exerts its therapeutic effect on steroid-induced INFH is through targeting exosomal miRNAs to regulate multiple signaling pathways such as PI3K-Akt signaling pathway, MAPK signaling pathway, and Wnt signaling pathway which is upstream and downstream of bone metabolism and bone reconstruction by up-regulating and down-regulating some miRNAs. In addition, this study analyzed for the first time the active ingredients of MQEF aqueous decoction extract by metabolomics to clarify the distribution of small and medium molecule compounds in MQEF in addition to clarifying their distribution pattern and proportion of small and medium molecules in MQEF. Together, bioinformatics analysis of the active ingredients in MQEF was conducted to clarify the possible targets and specific pathways of MQEF, which has a positive impact on the pharmacological research and molecular mechanism of MQEF. Further clinical and basic experimental studies are needed in the future.

## Data availability statement

The datasets presented in this study can be found in online repositories. The names of the repository/repositories and accession number(s) can be found in the article/**Supplementary Material**.

## Ethics statement

The animal study was reviewed and approved by the Institutional Animal Care and Use Committee at Tongji Medical College, Huazhong University of Science and Technology. [2020] IACUC Number: 2695.

## Author contributions

BS and WZ conceived the idea for the study and provided critical revision of the manuscript. BS, WZ, JL, FZ, and CM performed the experiments. JL, FZ, and CM participated in study design, data acquisition and data analysis. BS, WZ, and JL participated in writing and drafting of the manuscript. All authors read and approved the final manuscript.

## Funding

This study was partially funded by the National Natural Science Foundation of China (Project Number: 81974546, 82174182, 81974249, and 82004201).

## Acknowledgments

The authors would like to thank GLW (Beijing Genomics Institute) for his expertise and guidelines for performing the transcriptome sequencing. The authors would like to thank Editage (www.editage.cn) for English language editing.

## Conflict of interest

The authors declare that the research was conducted in the absence of any commercial or financial relationships that could be construed as a potential conflict of interest.

## Publisher’s note

All claims expressed in this article are solely those of the authors and do not necessarily represent those of their affiliated organizations, or those of the publisher, the editors and the reviewers. Any product that may be evaluated in this article, or claim that may be made by its manufacturer, is not guaranteed or endorsed by the publisher.

## Supplementary material

Supplementary Figure 1 The quality control of the extracted exosomes. **(A)** NTA was used to identify particle size and concentration of nanoparticles; **(B)** The expression of several exosome-specific markers CD63 and TSG101 was detected using Western blotting.

Supplementary Figure 2 miRNA expression level distribution. **(A)** Measured number of known and unknown genes. **(B)** Boxplots show the distribution of gene expression levels in each sample, and the disperse degree of the data distribution can be observed. **(C)** To visualize the number of genes in different TPM range of each sample, we performed statistics on the number of genes in three situations of TPM (TPM <= 1, TPM 1–10, TPM > = 10). **(D)** The density map can show the trend of gene abundance in the sample as the expression level changes, and it can clearly reflect the interval of gene expression concentration in the sample. **(E)** The number of transcription factors obtained from the multi-omics association analysis of the detected genes and transcription factors was ranked from high to low.

Supplementary Figure 3 Data filtering and quality control. **(A)** Reads filtering (small RNA). (**B**) N: The number of reads with an unknown base N content of less than 5% and the proportion of total raw reads; Adapter: the number of reads containing adapters (reads contaminated by the adapter) and the proportion in total raw reads; Low Quality: low-quality reads (The proportion of bases with a quality score below 15 to the total number of bases in the reads is less than 20% of the reads) and the proportion of the total raw reads; Clean Reads: the number of filtered reads and the proportion of the total raw reads. **(C)** The X-axis represents the position of the base in reads, and the Y-axis represents the content ratio of such bases. Under normal circumstances, the base content of each position of reads is stable without AT or GC separation. **(D)** The X-axis represents the position of the base in reads, and the Y-axis represents the quality score of the base. Each point in the figure represents the total number of bases at a certain quality score at this position. The darker the color is, the more the amount is. The distribution of base quality reflects the accuracy of sequencing reads. Sequencing platform, sequencing reagents, and sample quality all may affect base quality. Under normal circumstances, the quality of the first few bases in reads is not high, because the random primers do not bind the RNA template well during reverse transcription; as the sequencing length increases, the proportion of high-quality bases will increase. However, after the length reaches a certain threshold, the proportion of high-quality bases will decrease due to the consumption of sequencing reagents. On the whole, if the proportion of bases of low quality (quality <20) is low, it means that the sequencing quality is better. **(E)** small RNA classification. **(F)** The X-axis represents the small RNA length (the length of the sequenced fragment after removing the adaptor), and the Y-axis represents the length of small RNA as a percentage of all small RNAs. This figure shows the distribution of small RNA lengths. If the same small RNA is detected multiple times, it will be calculated multiple times.

Supplementary Figure 4 Extracted ion chromatograph. The MRM metabolites extraction chromatogram mass spectra (XIC; extracted ion chromatograph) of QC samples mixed with all samples, the horizontal coordinate is the retention time RT (retention time in min) for metabolite detection, and the vertical coordinate is the intensity of ion flow for metabolite target ion detection (intensity: the unit is cps [count per second]). Each mass spectrometry peak of a different color represents a metabolite detected.

Supplementary Table 1 597 predicted target genes. The Canonical SMILES numbers of the 34 active chemical components were entered into the Swiss Target Prediction database (http://www.swisstargetprediction.ch/) and their potential action targets against Homo sapiens were predicted according to their chemical structures.

Supplementary Table 2 354 genes in PI3K-Akt signaling pathway. All targets of PI3K-Akt signaling pathway were downloaded from the KEGG database (http://www.genome.jp/kegg/). A total of 354 pathway targets were obtained.

Supplementary Table 3 62 key targets for MQEF to exert anti-INFH through the PI3K-Akt signaling pathway. The intersection of 354 targets in the PI3K-Akt pathway with 597 targets in the MQEF were obtained by drawing Venn diagrams from the R package (Venn Diagram).
