# Supplementary figures and images for: The analysis of Modified Qing’ E Formula on the differential expression of exosomal miRNAs in the femoral head bone tissue of mice with steroid-induced ischemic necrosis of femoral head
Source: Front Endocrinol (Lausanne). 2022 Aug 10;13:954778. doi: 10.3389/fendo.2022.954778 (PMC9399624; doi:10.3389/fendo.2022.954778)

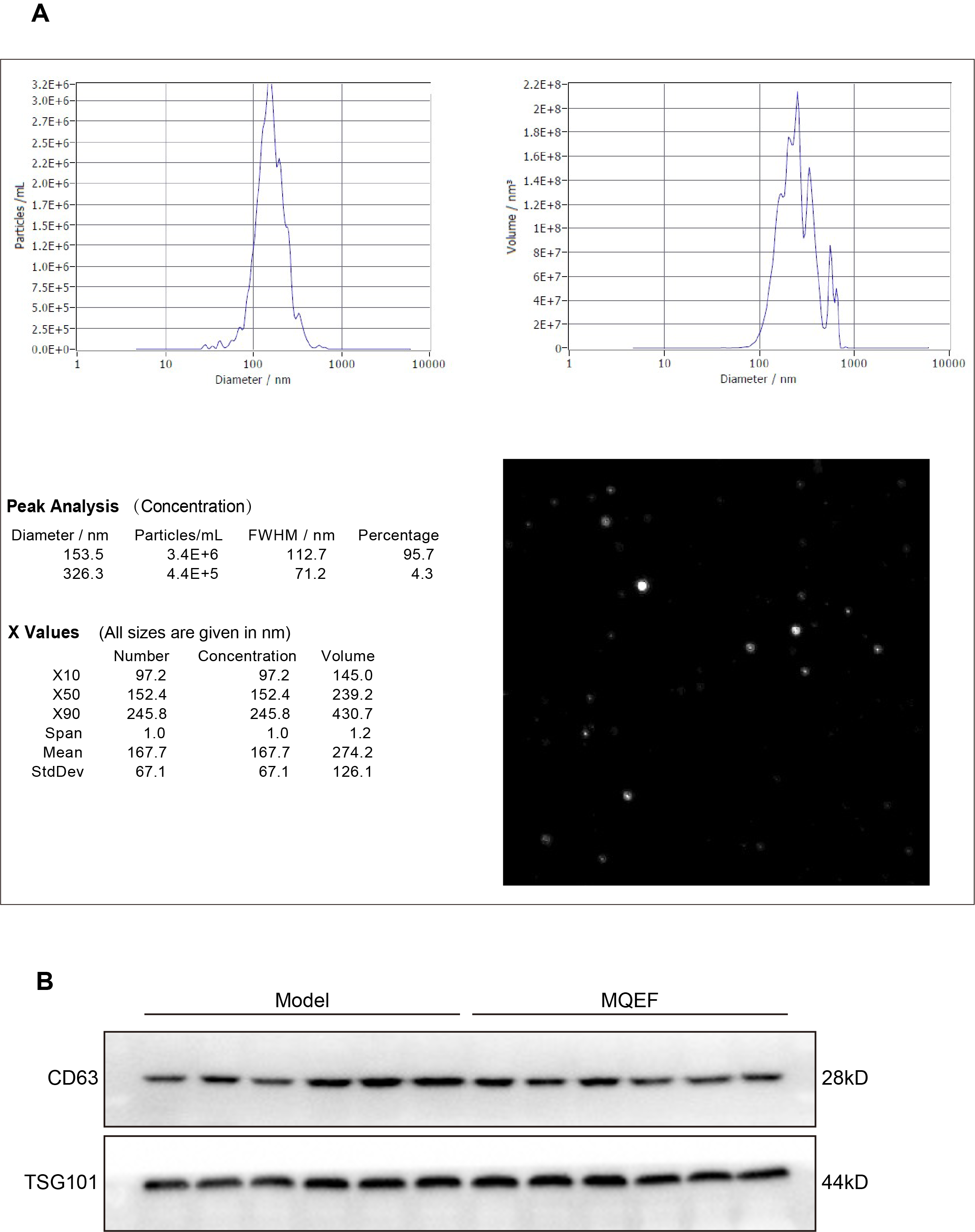

Supplement: Supplementary Figure 1 — The quality control of the extracted exosomes. (A) NTA was used to identify particle size and concentration of nanoparticles; (B) The expression of several exosome-specific markers CD63 and TSG101 was detected using Western blotting. [file Image_1.tif]

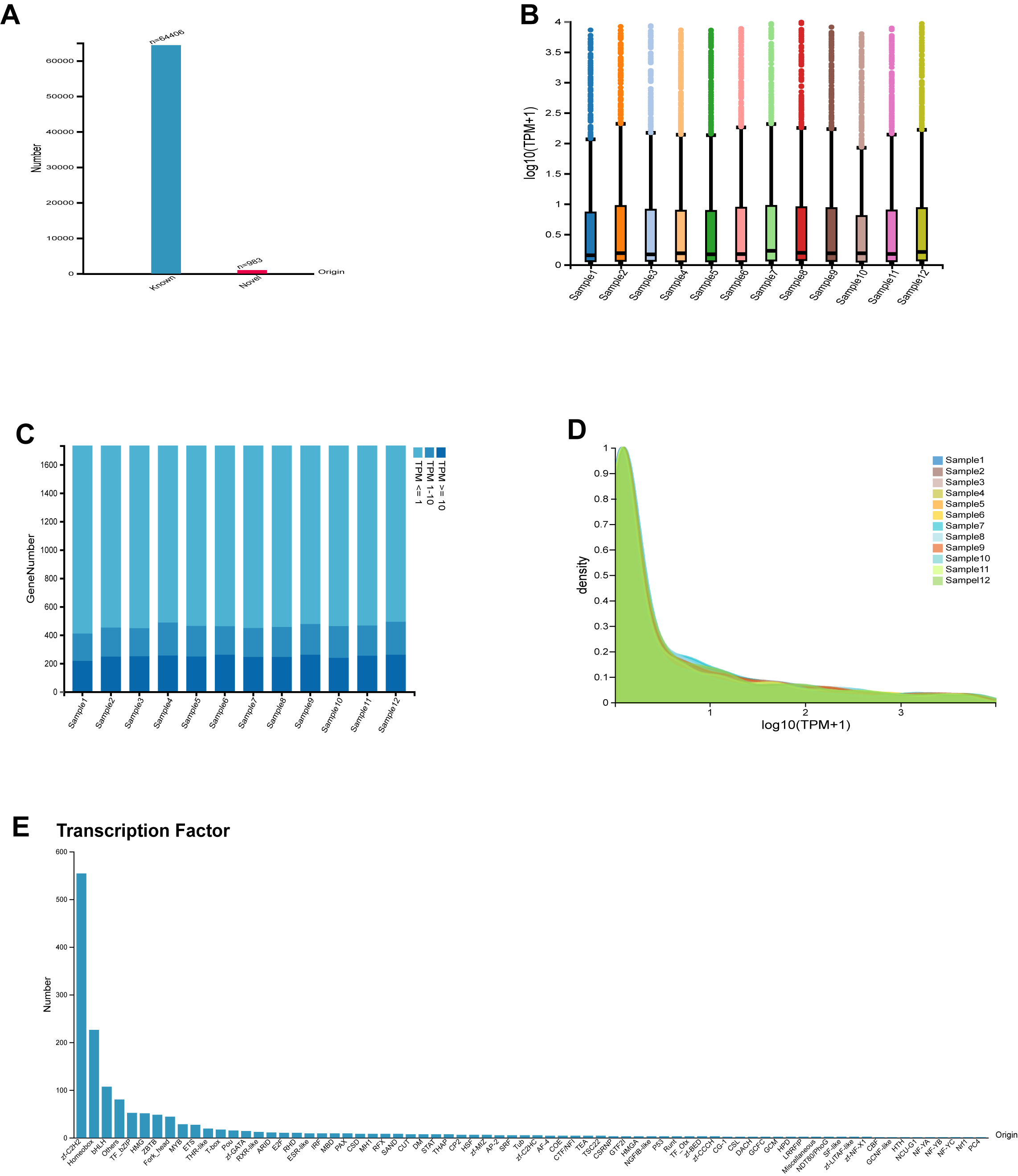

Supplement: Supplementary Figure 2 — miRNA expression level distribution. (A) Measured number of known and unknown genes. (B) Boxplots show the distribution of gene expression levels in each sample, and the disperse degree of the data distribution can be observed. (C) To visualize the number of genes in different TPM range of each sample, we performed statistics on the number of genes in three situations of TPM (TPM <= 1, TPM 1–10, TPM > = 10). (D) The density map can show the trend of gene abundance in the sample as the expression level changes, and it can clearly reflect the interval of gene expression concentration in the sample. (E) The number of transcription factors obtained from the multi-omics association analysis of the detected genes and transcription factors was ranked from high to low. [file Image_2.tif]

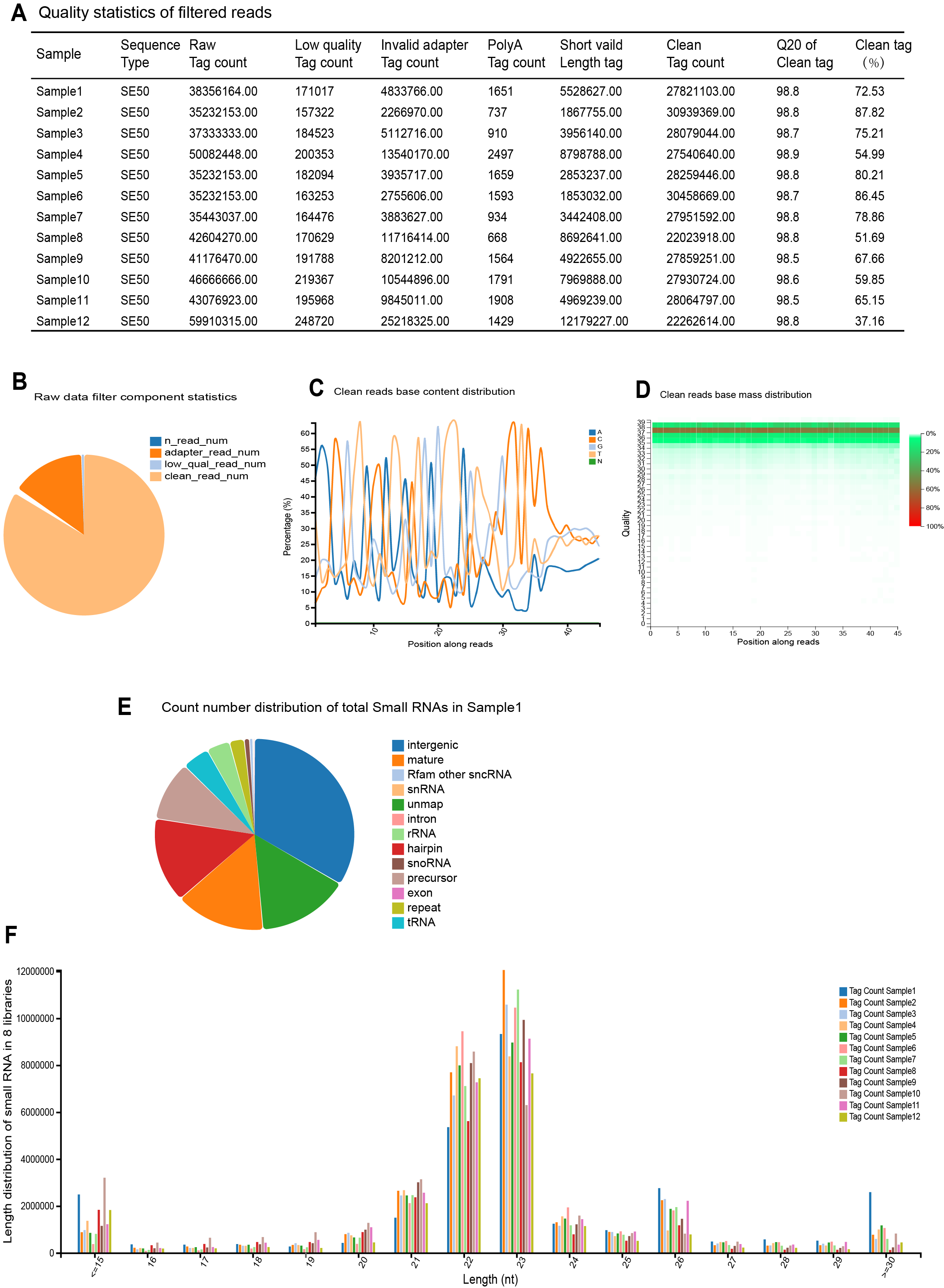

Supplement: Supplementary Figure 3 — Data filtering and quality control. (A) Reads filtering (small RNA). (B) N: The number of reads with an unknown base N content of less than 5% and the proportion of total raw reads; Adapter: the number of reads containing adapters (reads contaminated by the adapter) and the proportion in total raw reads; Low Quality: low-quality reads (The proportion of bases with a quality score below 15 to the total number of bases in the reads is less than 20% of the reads) and the proportion of the total raw reads; Clean Reads: the number of filtered reads and the proportion of the total raw reads. (C) The X-axis represents the position of the base in reads, and the Y-axis represents the content ratio of such bases. Under normal circumstances, the base content of each position of reads is stable without AT or GC separation. (D) The X-axis represents the position of the base in reads, and the Y-axis represents the quality score of the base. Each point in the figure represents the total number of bases at a certain quality score at this position. The darker the color is, the more the amount is. The distribution of base quality reflects the accuracy of sequencing reads. Sequencing platform, sequencing reagents, and sample quality all may affect base quality. Under normal circumstances, the quality of the first few bases in reads is not high, because the random primers do not bind the RNA template well during reverse transcription; as the sequencing length increases, the proportion of high-quality bases will increase. However, after the length reaches a certain threshold, the proportion of high-quality bases will decrease due to the consumption of sequencing reagents. On the whole, if the proportion of bases of low quality (quality <20) is low, it means that the sequencing quality is better. (E) small RNA classification. (F) The X-axis represents the small RNA length (the length of the sequenced fragment after removing the adaptor), and the Y-axis represents the length of small RNA [file Image_3.tif]

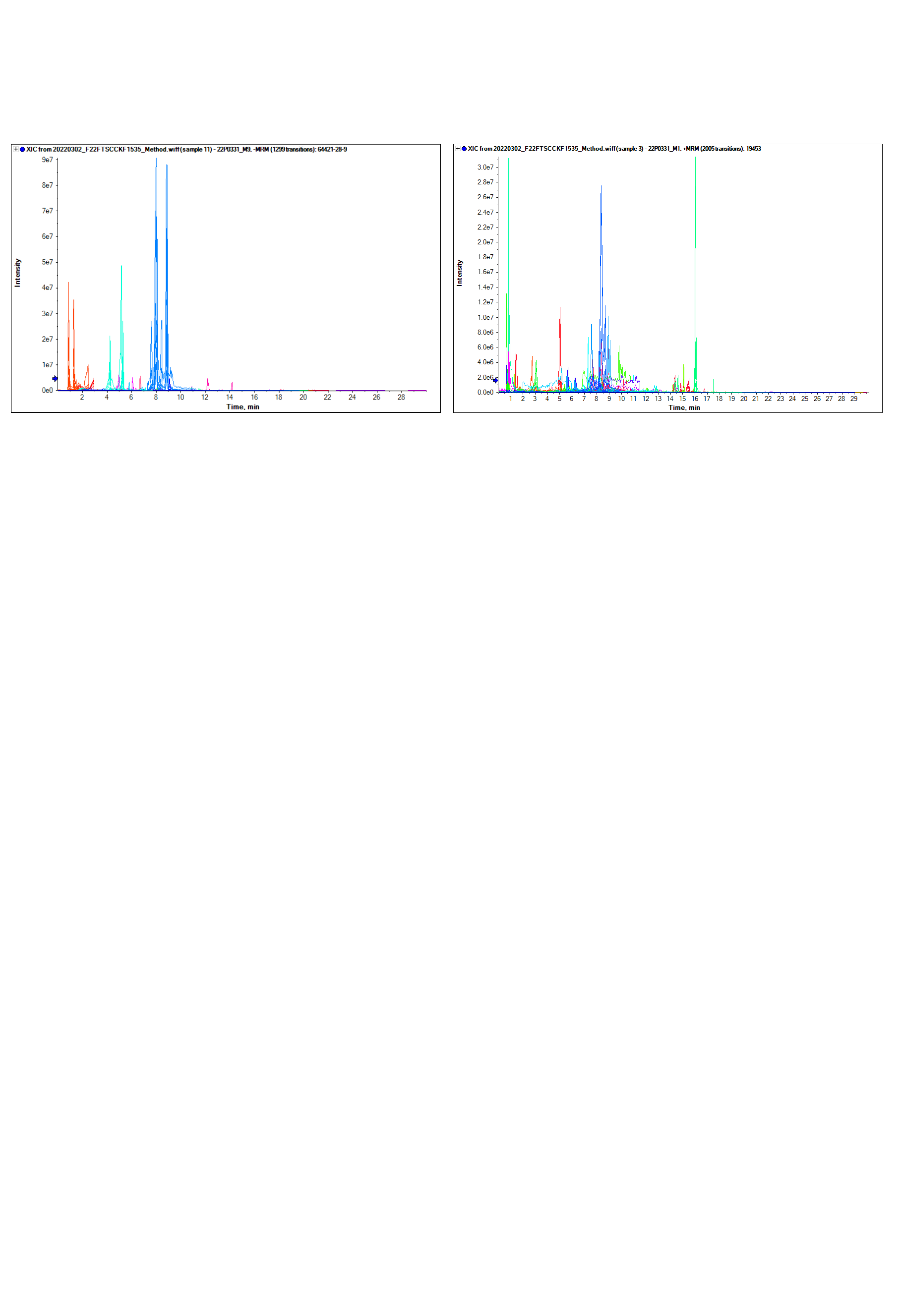

Supplement: Supplementary Figure 4 — Extracted ion chromatograph. The MRM metabolites extraction chromatogram mass spectra (XIC; extracted ion chromatograph) of QC samples mixed with all samples, the horizontal coordinate is the retention time RT (retention time in min) for metabolite detection, and the vertical coordinate is the intensity of ion flow for metabolite target ion detection (intensity: the unit is cps [count per second]). Each mass spectrometry peak of a different color represents a metabolite detected. [file Image_4.tiff]
